# Supplementary material for: TRIM52 maintains cellular fitness and is under tight proteolytic control by multiple giant E3 ligases
Source: Nat Commun. 2025 Apr 24;16:3894. doi: 10.1038/s41467-025-59129-y (PMC12022042; doi:10.1038/s41467-025-59129-y)
Supplement: Supplementary file 1 — Supplementary Information [file 41467_2025_59129_MOESM1_ESM.pdf]

<u>**Supplementary information**</u>

Supplementary figures and legends

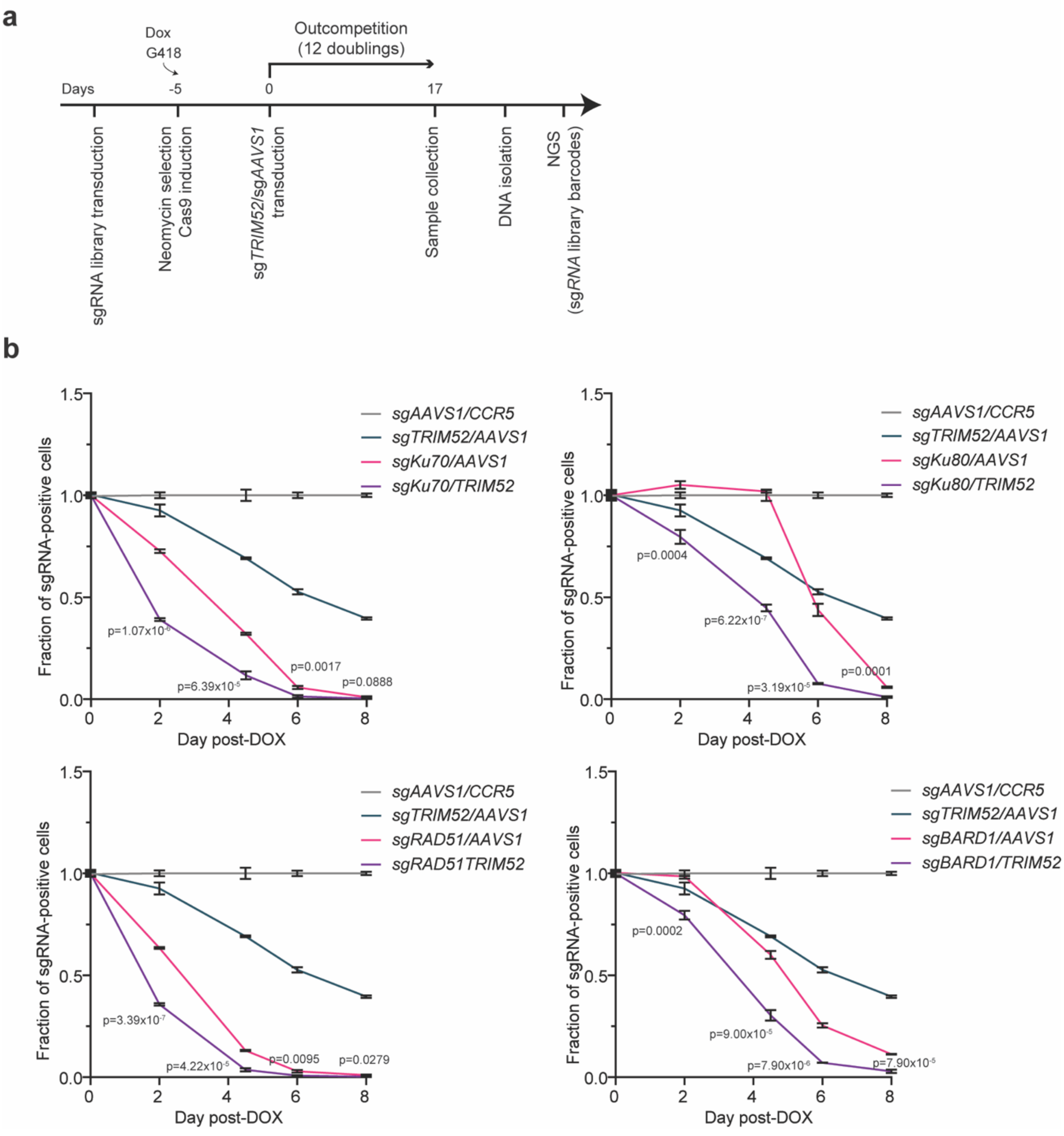

Supplementary figure 1 – TRIM52 is required for cell fitness. (**a**) Timeline of the genetic modifier screen. (**b**) Relative cell fitness of single sg*TRIM52*, *sgKu70, sgKu80, sgRAD51,* and double-targeted cells compared to untransduced cells was determined by measuring the percentage of iRFP fluorescent cells over the indicated time period. sgRNA transduced cells were normalized to safe-harbour locus sg*AAVS1/CCR5*-targeted cells, relative to untransduced cells. Data represent biological replicates, n = 3. Single and double KO samples were analysed by multiple unpaired t-tests. ns: $p > 0.05$, *: $p < 0.05$, **: $p < 0.01$, ***: $p < 0.001$.

Data represent means. Error bars indicate standard deviations). Source data are provided as a Source Data file.

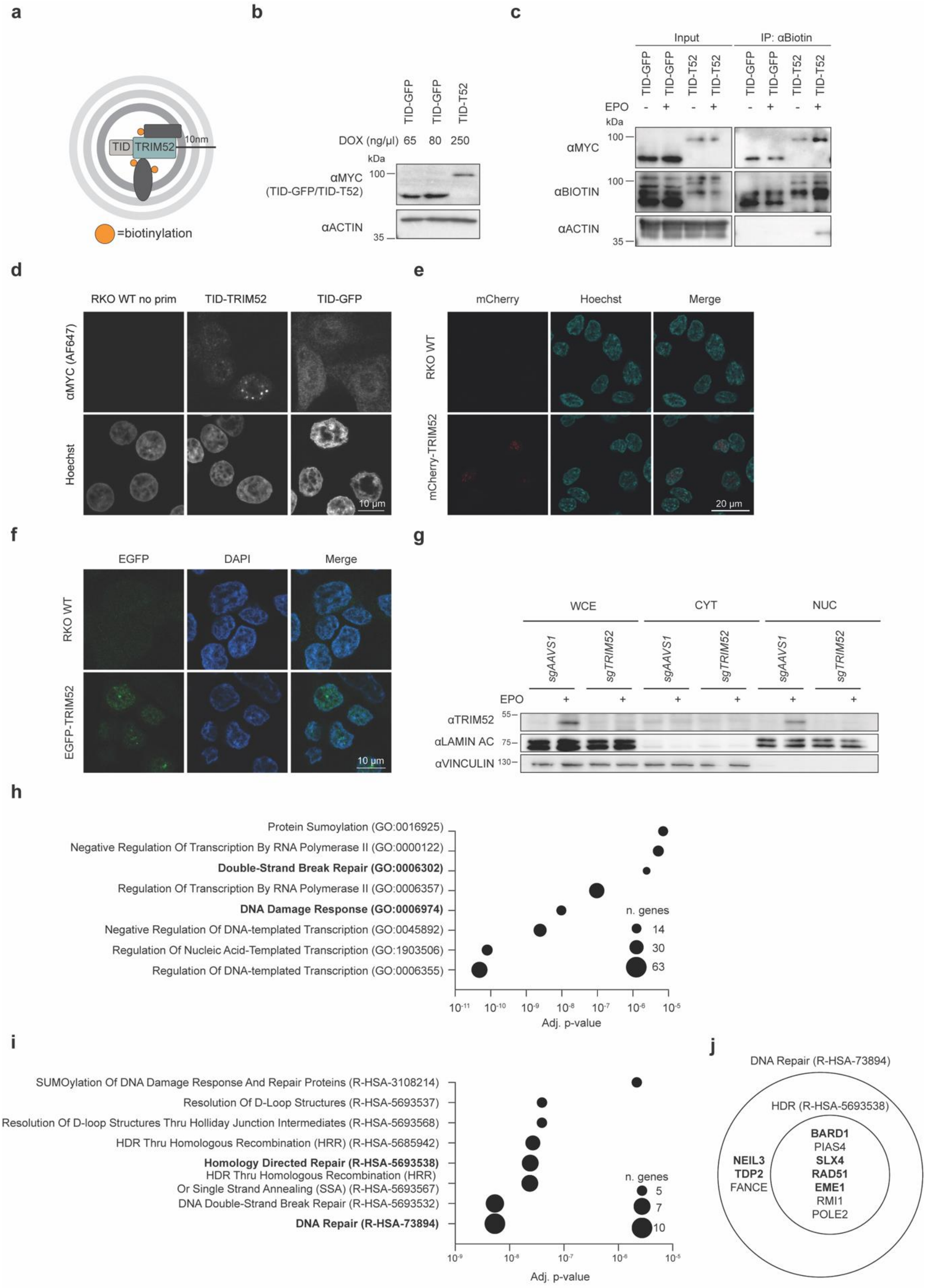
a
b
c
TID
TRIM52
10nm
=biotinylation
TID-GFP
TID-GFP
TID-T52
DOX (ng/µl) 65 80 250
kDa
αMYC
(TID-GFP/TID-T52)
100
αACTIN
35
Input
IP: αBiotin
TID-GFP
TID-GFP
TID-T52
TID-T52
EPO - + - +
αMYC
αBIOTIN
αACTIN
d
RKO WT no prim
TID-TRIM52
TID-GFP
αMYC (AF647)
Hoechst
10 µm
e
mCherry
Hoechst
Merge
RKO WT
mCherry-TRIM52
20 µm
f
EGFP
DAPI
Merge
RKO WT
EGFP-TRIM52
10 µm
g
WCE
CYT
NUC
sgAAVS1
sgTRIM52
EPO
αTRIM52
αLAMIN AC
αVINCULIN
55
75
130
h
Protein Sumoylation (GO:0016925)
Negative Regulation Of Transcription By RNA Polymerase II (GO:0000122)
Double-Strand Break Repair (GO:0006302)
Regulation Of Transcription By RNA Polymerase II (GO:0006357)
DNA Damage Response (GO:0006974)
Negative Regulation Of DNA-templated Transcription (GO:0045892)
Regulation Of Nucleic Acid-Templated Transcription (GO:1903506)
Regulation Of DNA-templated Transcription (GO:0006355)
n. genes
14
30
63
Adj. p-value
i
SUMOylation Of DNA Damage Response And Repair Proteins (R-HSA-3108214)
Resolution Of D-Loop Structures (R-HSA-5693537)
Resolution Of D-loop Structures Thru Holliday Junction Intermediates (R-HSA-5693568)
HDR Thru Homologous Recombination (HRR) (R-HSA-5685942)
Homology Directed Repair (R-HSA-5693538)
HDR Thru Homologous Recombination (HRR)
Or Single Strand Annealing (SSA) (R-HSA-5693567)
DNA Double-Strand Break Repair (R-HSA-5693532)
DNA Repair (R-HSA-73894)
n. genes
5
7
10
Adj. p-value
j
DNA Repair (R-HSA-73894)
HDR (R-HSA-5693538)
BARD1
PIAS4
SLX4
RAD51
EME1
RMI1
POLE2
NEIL3
TDP2
FANCE

Supplementary figure 2 – TRIM52 interacts with the DNA repair machinery. (**a**) Schematic representation of TurboID proximity labelling principle. **(b)** RKO cells harbouring Dox-inducible TurboID constructs were treated with the indicated concentrations of Dox, and protein expression analysed by WB at 48 h post-induction. (**c**) RKO cells expressing TurboID-TRIM52 or TurboID-EGFP fusion proteins were treated with epoxomicin for 5 h. and biotin for the last 15 min. Proteins were streptavidin affinity purified and analysed by WB. (**d**) RKO cells stably expressing TurboID-TRIM52 and TurboID-EGFP were treated with epoxomicin for 5 h., fixed, and their subcellular localization determined by immunofluorescence confocal microscopy. (**e-f**) RKO cells stably expressing **e)** mCherry-TRIM52, or **f)** EGFP-TRIM52 were treated with epoxomicin for 5 h., fixed, and their subcellular localization determined by immunofluorescence confocal microscopy. (**g**) RKO cells harbouring Dox-inducible Cas9 were transduced with sgRNA vectors targeting *TRIM52* or the safe-harbor locus *AAVS1*. Cas9 was induced by Dox treatment for 5 days. Cells were treated with epoxomicin (EPO) for 5 h., whole cell extracts (WCE), cytoplasmic (CYT) and nuclear (NUC) fractions extracted and analysed by WB. (**h**) Putative interactors of TRIM52 with p-value < 0.05 and Log2 fold change > 2.5 were selected and analysed by gene ontology enrichment analysis. The enriched GO terms are plotted based on their adjusted p-value and the number of genes within each GO Biological Processes term. (**i**) Genes included in the DNA Damage Response (GO:0006974) and Double-strand Break Repair (GO:0006302) GO terms were further analysed against the Reactome 2022 database. The enriched pathways were plotted based on their adjusted p-value and the number of genes within each Reactome 2022 term. (**j**) Venn-diagram describing the putative TRIM52 interactors in the DNA repair (R-HSA-73894) and HDR (R-HSA-5693538) GO terms. Source data are provided as a Source Data file.

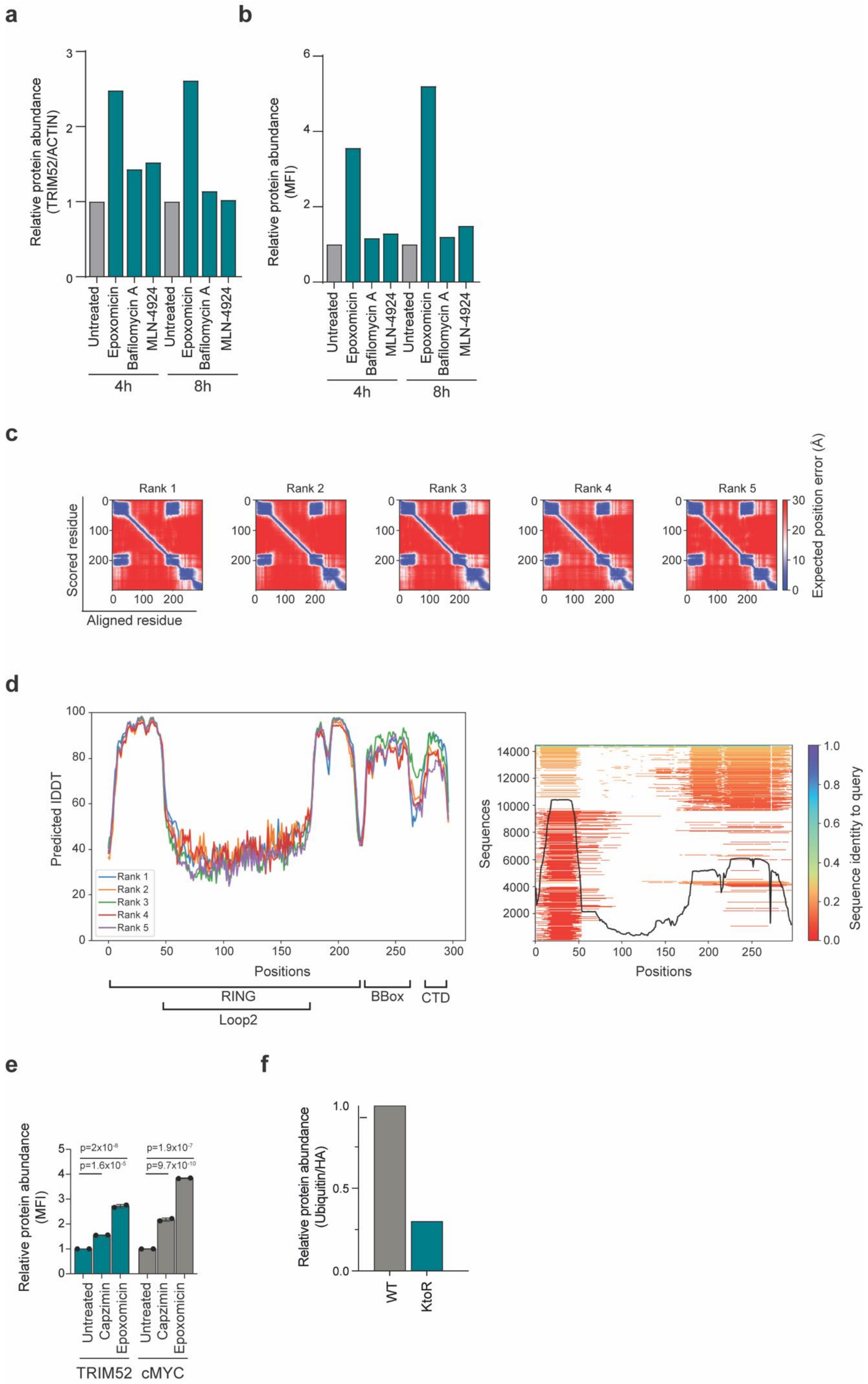
a
Relative protein abundance (TRIM52/ACTIN)
Untreated
Epoxomicin
Bafilomycin A
MLN-4924
4h
8h
b
Relative protein abundance (MFI)
c
Rank 1
Rank 2
Rank 3
Rank 4
Rank 5
Scored residue
Aligned residue
Expected position error (Å)
d
Predicted lDDT
Positions
RING
Loop2
BBox
CTD
Sequences
Sequence identity to query
e
p=2x10-8
p=1.6x10-5
p=1.9x10-7
p=9.7x10-10
Capzimin
TRIM52
cMYC
f
Relative protein abundance (Ubiquitin/HA)
WT
KtoR

Supplementary figure 3 – TRIM52 is degraded by the ubiquitin-proteasome system. (**a**) RKO cells expressing Ollas-tagged EGFP-TRIM52 were treated for 4 or 8 h. with epoxomicin, bafilomycin A, or MLN-4924. Ollas-EGFP-TRIM52 protein levels determined by WB, quantified and normalized to actin, or (**b**) by flow cytometry, their MFI quantified, normalized to MYC-mCherry and the untreated control, and plotted. (**c**) Predicted aligned error plot of the AlphaFold2 model for TRIM52 structure prediction. (**d**) Predicted lDDT per position and sequence coverage of the AlphaFold2 model for TRIM52 structure prediction. (**e**) RKO cells expressing EGFP-TRIM52 or mCherry-cMYC fusion proteins were treated for 5 h. with the 20S proteasome inhibitor epoxomicin, or 19S-specific inhibitor capzimin. EGFP-TRIM52 and mCherry-cMYC protein levels were determined by flow cytometry, and MFI quantified. Data represent biological replicates, n = 2. Data were analysed by 1-way ANOVA. ns: $p > 0.05$, *: $p < 0.05$, **: $p < 0.01$, ***: $p < 0.001$. Data represent means. Error bars indicate standard deviations. (**f**) HEK-293T cells expressing HA-tagged WT TRIM52 or a lysine-to-arginine mutant were treated with epoxomicin for 5 h., after which TRIM52 was immunoprecipitated, and its ubiquitination analysed by WB. The ubiquitin signal was quantified and normalized to WT HA-TRIM52 levels. Source data are provided as a Source Data file.

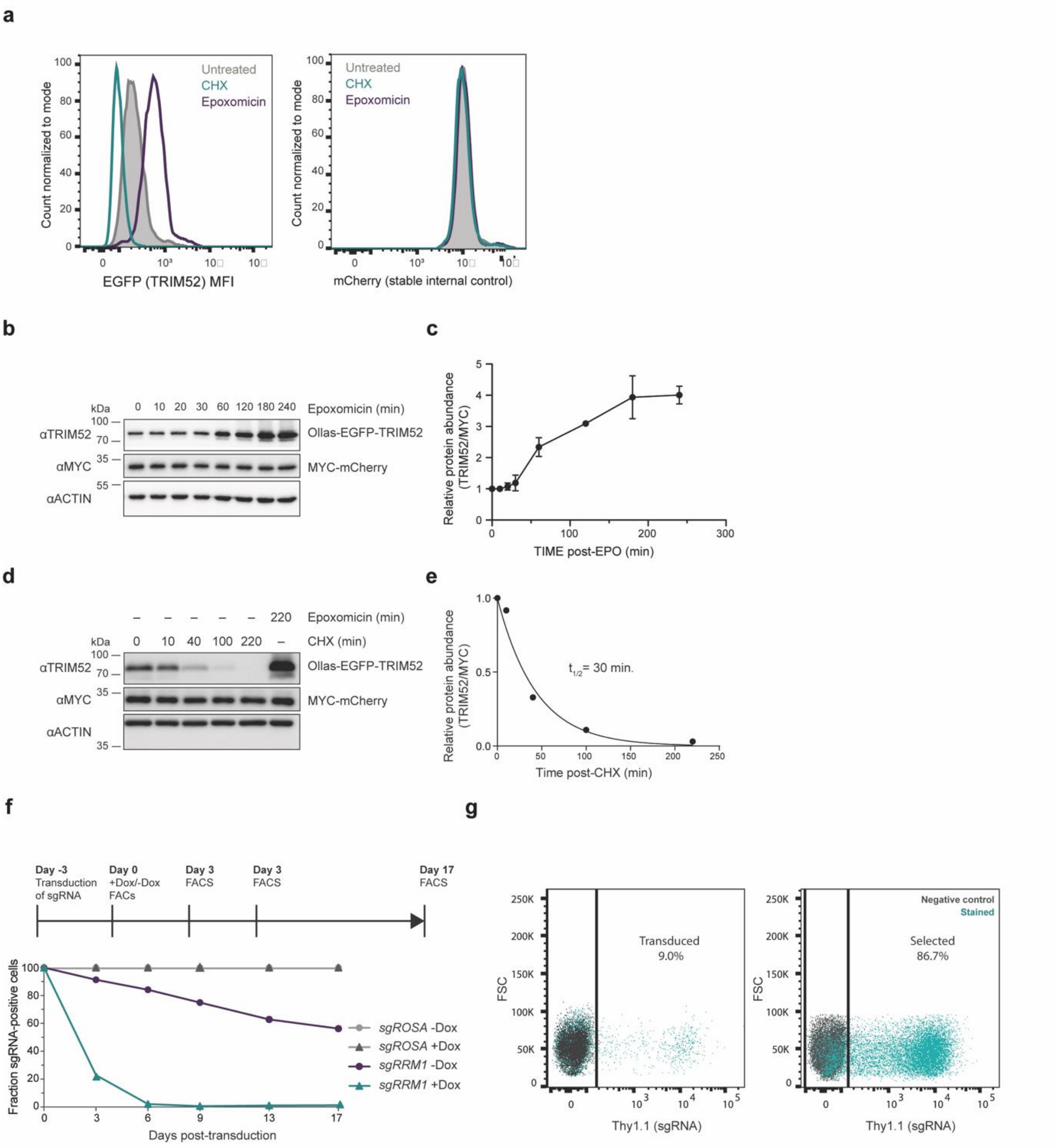

Supplementary figure 4 - TRIM52 is targeted for degradation by multiple giant E3 ligases. (**a**) RKO cells were stably transduced with the TRIM52 reporter construct and single cell sorted based on mCherry and EGFP fluorescence intensities. The selected screening cell clone was expanded and treated for 4 h. with the proteasome inhibitor epoxomicin, or CHX. Ollas-EGFP-TRIM52 and MYC-mCherry protein levels were quantified by flow cytometry. (**b**) RKO cells stably transduced with the TRIM52 reporter construct were treated with epoxomicin for the indicated time periods. WCEs were analysed by WB, (**c**) quantified and plotted. Data represent biological replicates, n = 3. Data represent means. Error bars indicate standard

deviations. (**d**) RKO cells stably transduced with the TRIM52 reporter construct were treated with epoxomicin or CHX for the indicated times. WCEs were analysed by WB, (**e**) quantified and single-exponential decay curves were fitted to calculate half-life. (**f**) The screening cell line was transduced with sgRNAs targeting the essential gene *RRM1* or the *hROSA* safe harbor locus. Cells were mixed with WT cells and their cell fitness expressed as relative iRFP-positive fraction in the cell pool. (**g**) 4 days after transduction of the screening cell line with a lentiviral sgRNA library, the percentage of transduced cells was determined by analysing the expression of Thy1.1 using flow cytometry. Transduced cells were selected using G418 for 4 days and Thy1.1 expression was analysed using flow cytometry. Source data are provided as a Source Data file.

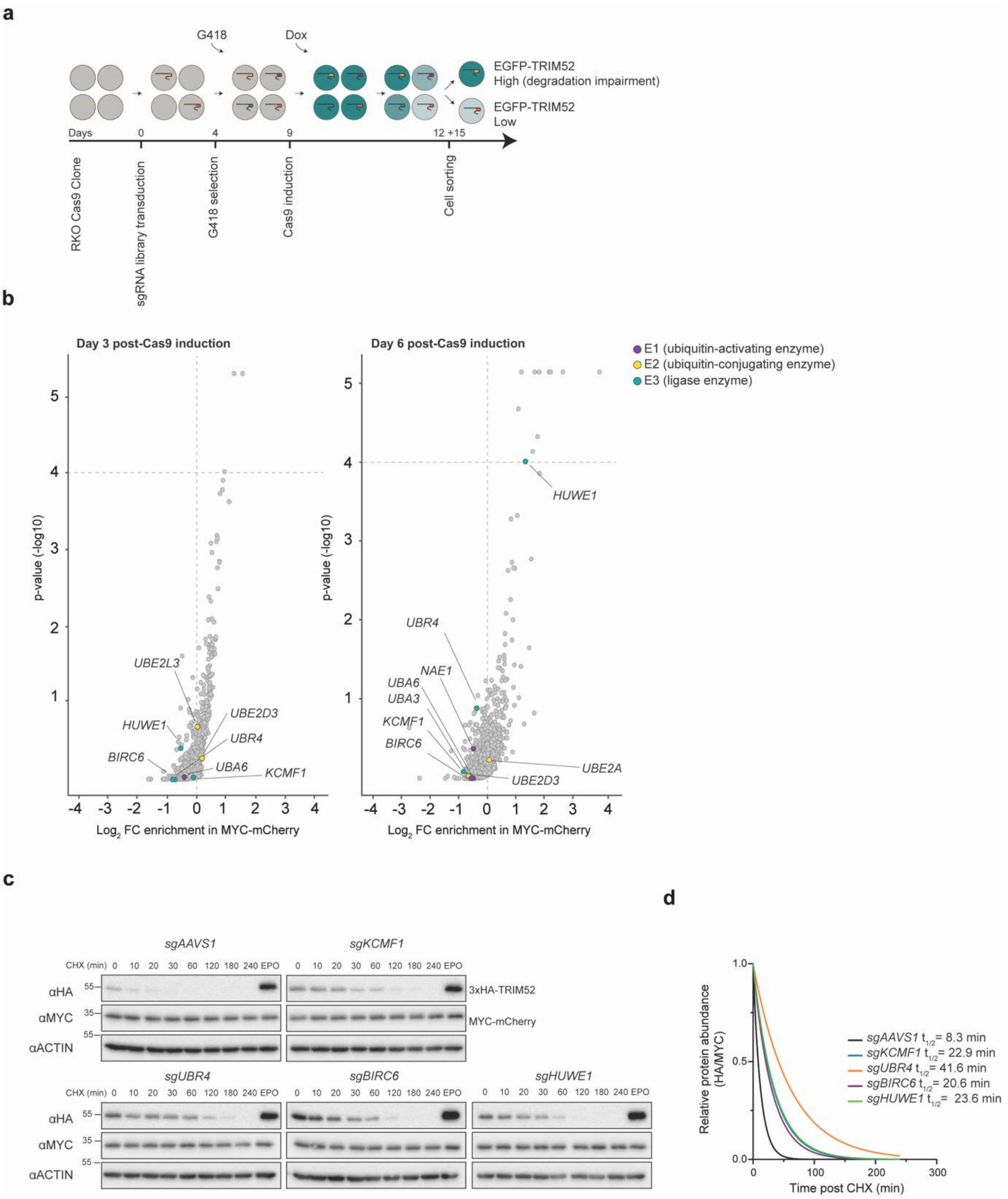

Supplementary figure 5 - TRIM52 is targeted for degradation by multiple giant E3 ligases. (**a**) Schematic representation of FACS-based screen, grey and blue circles represent cells transduced with sgRNA vectors. (**b**) Enrichment plot of screen hits in the mCherry^{high} control sorted cell pool. (**c**) RKO cells stably expressing MYC-mCherry-P2A-HA-TRIM52 were treated with epoxomicin or CHX for the indicated time periods, after which WCEs were analysed by WB, (**d**) quantified, and single-exponential decay curves were fitted to calculate half-lives. Source data are provided as a Source Data file.

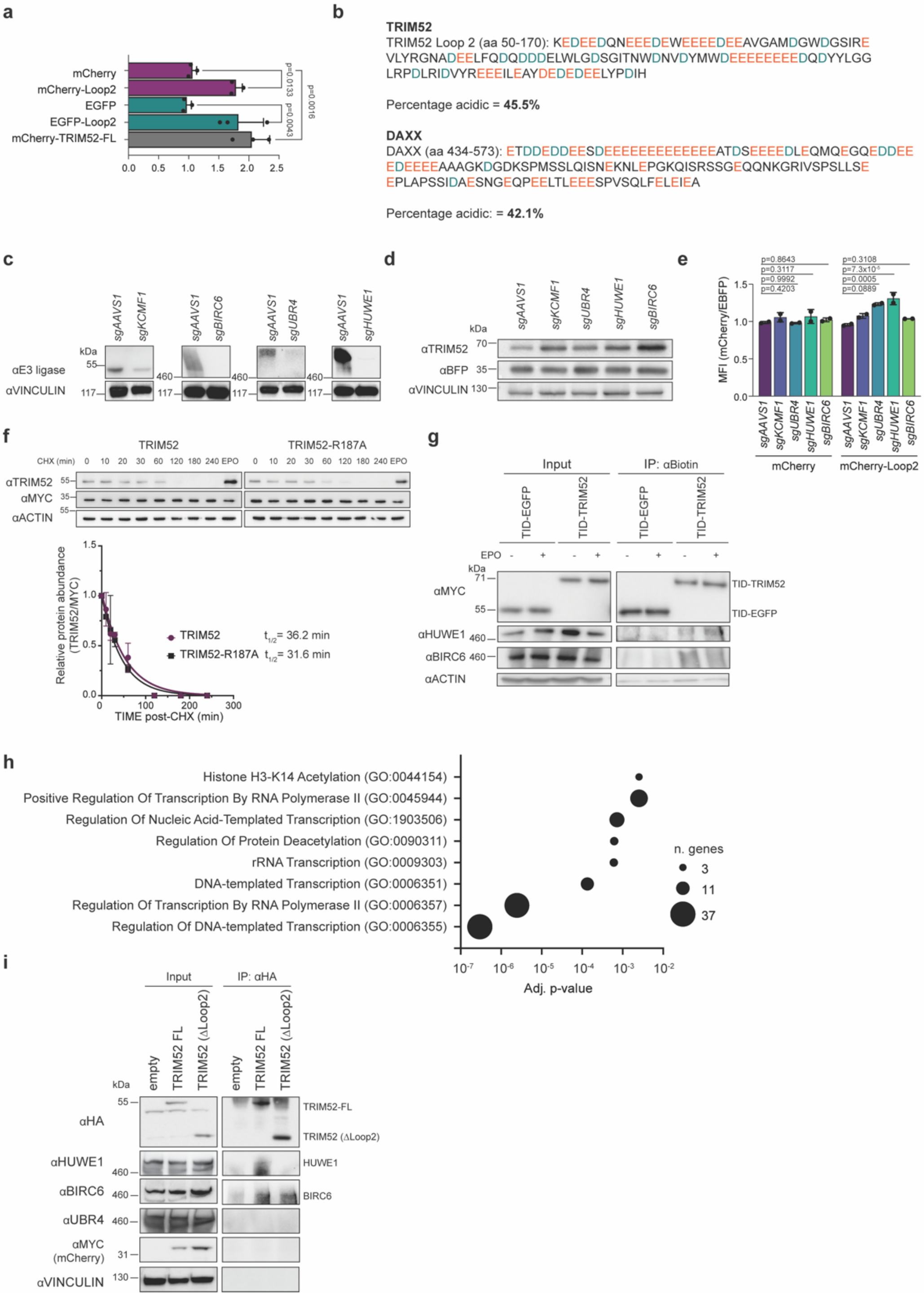
a
mCherry
mCherry-Loop2
EGFP
EGFP-Loop2
mCherry-TRIM52-FL
p=0.0133
p=0.0043
p=0.0016
b
TRIM52
TRIM52 Loop 2 (aa 50-170): KEDEEDQNEEEDEWEEEEDEEAVGAMDGWDGSIRE VLYRGNADEELFQDQDDDELWLGDSGITNWDNVDYMWDEEEEEEEEDQDYYLGG LRPDLRIDVYREEEILEAYDEDEDEELYPDIH
Percentage acidic = 45.5%
DAXX
DAXX (aa 434-573): ETDDEDDEESDEEEEEEEEEEEEEATDSEEEEDLEQMQEGQEDDEE EDEEEEAAAGKDGDKSPMSSLQISNEKNLEPGKQISRSSGEQQNKGRIVSPSLLSE EPLAPSSIDAESNGEQPEELTLEEESPVSQLFELEIEA
Percentage acidic: = 42.1%
c
sgAAVS1
sgKCMF1
sgBIRC6
sgUBR4
sgHUWE1
αE3 ligase
αVINCULIN
d
αTRIM52
αBFP
αVINCULIN
e
MFI (mCherry/EBFP)
mCherry
mCherry-Loop2
f
TRIM52
TRIM52-R187A
CHX (min)
αTRIM52
αMYC
αACTIN
Relative protein abundance (TRIM52/MYC)
TIME post-CHX (min)
TRIM52 $t_{1/2}$ = 36.2 min
TRIM52-R187A $t_{1/2}$ = 31.6 min
g
Input
IP: αBiotin
TID-EGFP
TID-TRIM52
EPO
αMYC
αHUWE1
αBIRC6
αACTIN
h
Histone H3-K14 Acetylation (GO:0044154)
Positive Regulation Of Transcription By RNA Polymerase II (GO:0045944)
Regulation Of Nucleic Acid-Templated Transcription (GO:1903506)
Regulation Of Protein Deacetylation (GO:0090311)
rRNA Transcription (GO:0009303)
DNA-templated Transcription (GO:0006351)
Regulation Of Transcription By RNA Polymerase II (GO:0006357)
Regulation Of DNA-templated Transcription (GO:0006355)
n. genes
3
11
37
Adj. p-value
i
Input
IP: αHA
empty
TRIM52 FL
TRIM52 (ΔLoop2)
αHA
αHUWE1
αBIRC6
αUBR4
αMYC (mCherry)
αVINCULIN
TRIM52-FL
TRIM52 (ΔLoop2)
HUWE1
BIRC6

Supplementary figure 6- BIRC6, HUWE1, and UBR4/KCMF1 target the extended loop 2 region in the TRIM52 RING domain. (**a**) HEK-293T cells expressing the indicated TwinStrep-mCherry- or TwinStrep-EGFP-tagged proteins were treated with epoxomicin for 5 h., after which protein levels were quantified by flow cytometry, normalized to internal control EBFP levels, and plotted relative to corresponding untreated samples. Data represent biological replicates, n = 3. Data were analysed by 1-way ANOVA. ns: $p > 0.05$, *: $p < 0.05$, **: $p < 0.01$, ***: $p < 0.001$, ****: $p < 0.0001$. Data represent means. Error bars indicate standard deviations. (**b**) Sequences of acidic residue-rich regions in the loop 2 region of TRIM52 and DAXX. (**c**) HEK-293T cells constitutively expressing Cas9 and sgRNAs targeting the indicated genes were analyzed by WB. (**d**) HEK-293T cells constitutively expressing Cas9 and sgRNA targeting the indicated E3 ligases were transfected with plasmids expressing TwinStrep-mCherry-tagged TRIM52. Protein levels were analysed by WB. (**e**) HEK-293T cells were transfected with plasmids expressing TwinStrep-mCherry or TwinStrep-mCherry-Loop2. Protein levels were measured by flow cytometry, normalized to the EBFP internal control and the sg*AAVS1* control, and plotted. Data represent biological replicates, n = 2. Data were analyzed by 2-way ANOVA. ns: $p > 0.05$, *: $p < 0.05$, **: $p < 0.01$, ***: $p < 0.001$, ****: $p < 0.0001$. Data represent means. Error bars indicate standard deviations. (**f**) RKO cells stably expressing TRIM52 or its catalytically inactive TRIM52-R187A mutant were treated with EPO or CHX for the indicated times. WCEs were analysed by WB, quantified and single-exponential decay curves were fitted to calculate half-life. Data represent biological replicates, n = 2. Error bars indicate standard deviations. (**g**) RKO cells expressing TurboID-TRIM52 or TurboID-EGFP fusion proteins were treated with biotin for 15 min., modified proteins were purified, and analysed by WB. (**h**) Putative interactors of TRIM52 with p-value < 0.05 and Log2 fold change > 2.5 were analysed for GO enrichment and plotted based on their adjusted p-value and the number of genes within each GO Biological Processes term. (**i**) HEK-293T cells expressing 3xHA-tagged TRIM52 were treated with epoxomicin for 5 h., after which TRIM52 was immunoprecipitated, and analysed by WB for co-immunoprecipitation with HUWE1, BIRC6, and UBR4. Source data are provided as a Source Data file.

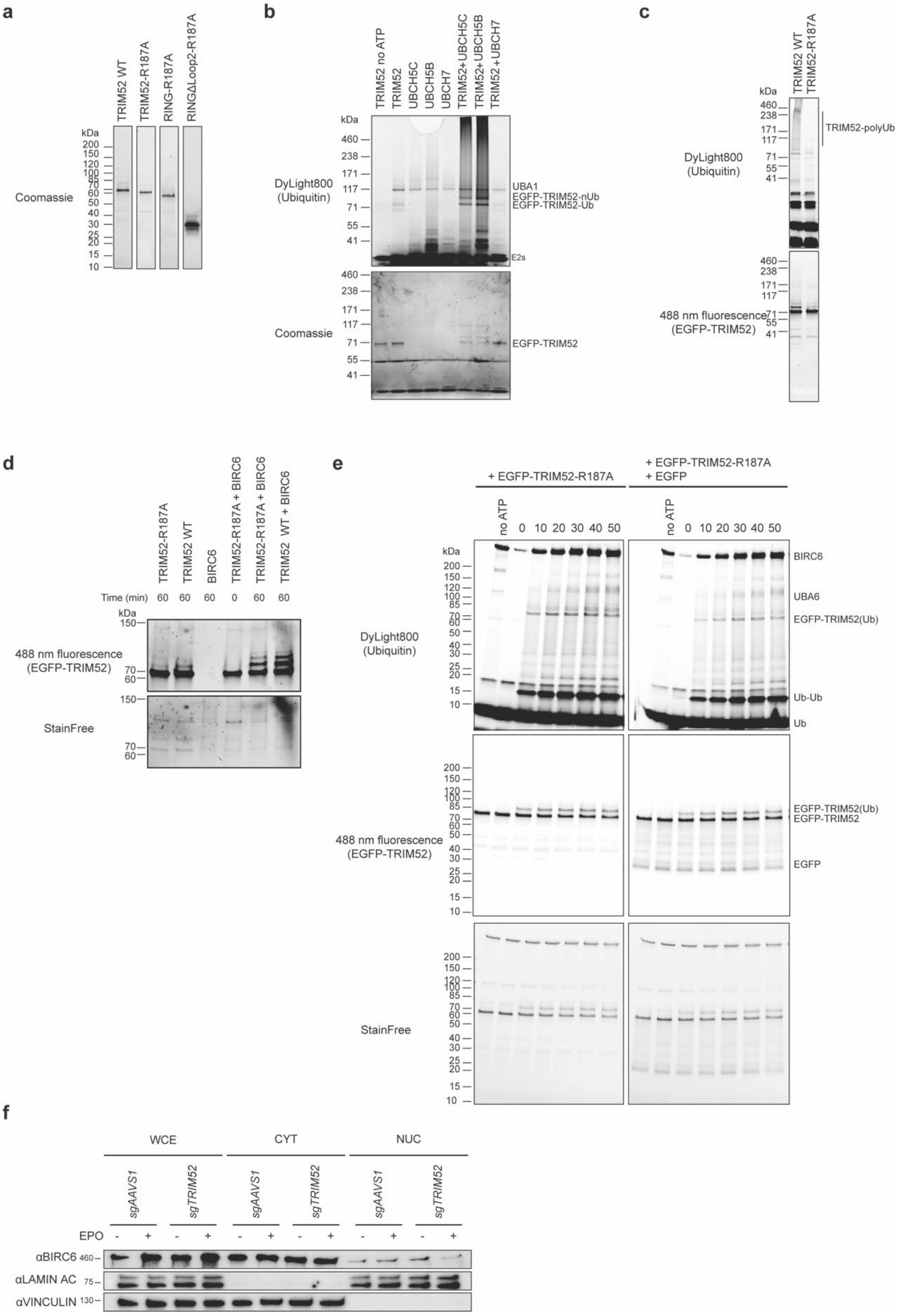

a
TRIM52 WT
TRIM52-R187A
RING-R187A
RINGΔLoop2-R187A
kDa
200
150
120
100
85
70
60
50
40
30
25
20
15
10
Coomassie
b
TRIM52 no ATP
TRIM52
UBCH5C
UBCH5B
UBCH7
TRIM52+UBCH5C
TRIM52+UBCH5B
TRIM52 + UBCH7
kDa
460
238
171
117
71
55
41
DyLight800 (Ubiquitin)
UBA1
EGFP-TRIM52-nUb
EGFP-TRIM52-Ub
E2s
Coomassie
EGFP-TRIM52
c
TRIM52 WT
TRIM52-R187A
kDa
460
238
171
117
71
55
41
DyLight800 (Ubiquitin)
TRIM52-polyUb
488 nm fluorescence (EGFP-TRIM52)
d
TRIM52-R187A
TRIM52 WT
BIRC6
TRIM52-R187A + BIRC6
TRIM52-R187A + BIRC6
TRIM52 WT + BIRC6
Time (min) 60 60 60 0 60 60
kDa
150
70
60
488 nm fluorescence (EGFP-TRIM52)
StainFree
e
+ EGFP-TRIM52-R187A
+ EGFP-TRIM52-R187A
+ EGFP
no ATP
0 10 20 30 40 50
kDa
200
150
120
100
85
70
60
50
40
30
25
20
15
10
DyLight800 (Ubiquitin)
BIRC6
UBA6
EGFP-TRIM52(Ub)
Ub-Ub
Ub
488 nm fluorescence (EGFP-TRIM52)
EGFP-TRIM52(Ub)
EGFP-TRIM52
EGFP
StainFree
f
WCE
CYT
NUC
sgAAVS1
sgTRIM52
EPO - + - + - + - + - + - +
αBIRC6 460
αLAMIN AC 75
αVINCULIN 130

Supplementary figure 7 - BIRC6 and UBR4/KCMF1 ubiquitinate TRIM52 in vitro. (**a**) TwinStrep-EGFP-tagged WT TRIM52, TRIM52-R187A, RING-R187A, and RINGΔLoop2-R187A were expressed in Hi5 insect cells, recombinant protein purified by streptavidin affinity matrix and size-exclusion chromatography and analysed by SDS-PAGE with Coomassie staining. (**b**) Ubiquitination assay of WT EGFP-TRIM52 with different E2s, UBA1, and DyLight800-labeled ubiquitin. The reactions were incubated for 1 h. at 37 °C in the presence of ATP. (**c**) Ubiquitination assay of WT EGFP-TRIM52 and its linchpin mutant (R187A) with UBCH5B, UBA1 and DyLight800-labeled ubiquitin. The reactions were incubated for 1h. at 37 °C in the presence of ATP. (**d**) Time-course ubiquitination assay of WT EGFP-TRIM52 and TRIM52-R187A with BIRC6, UBA6, and WT ubiquitin. The reactions were incubated for the indicated times at 37 °C in the presence of ATP. (**e**) Time-course ubiquitination assay of EGFP-TRIM52-R187A and EGFP as a control with BIRC6, UBA6, and DyLight800-labeled ubiquitin. The reactions were incubated for the indicated times at 37 °C in the presence of ATP. (**f**) RKO cells harbouring Dox-inducible Cas9 were transduced with sgRNA vectors targeting *TRIM52* or the safe-harbor locus *AAVS1*. Cas9 was induced by Dox treatment for 5 days. Cells were treated with epoxomicin for 5 h., whole cell extracts (WCE), cytoplasmic (CYT) and nuclear (NUC) fractions extracted and analysed by WB. Same samples as in Extended Fig. 2g, analysed on a 4-20% gradient gel. Source data are provided as a Source Data file.

Genetic screen gating strategy

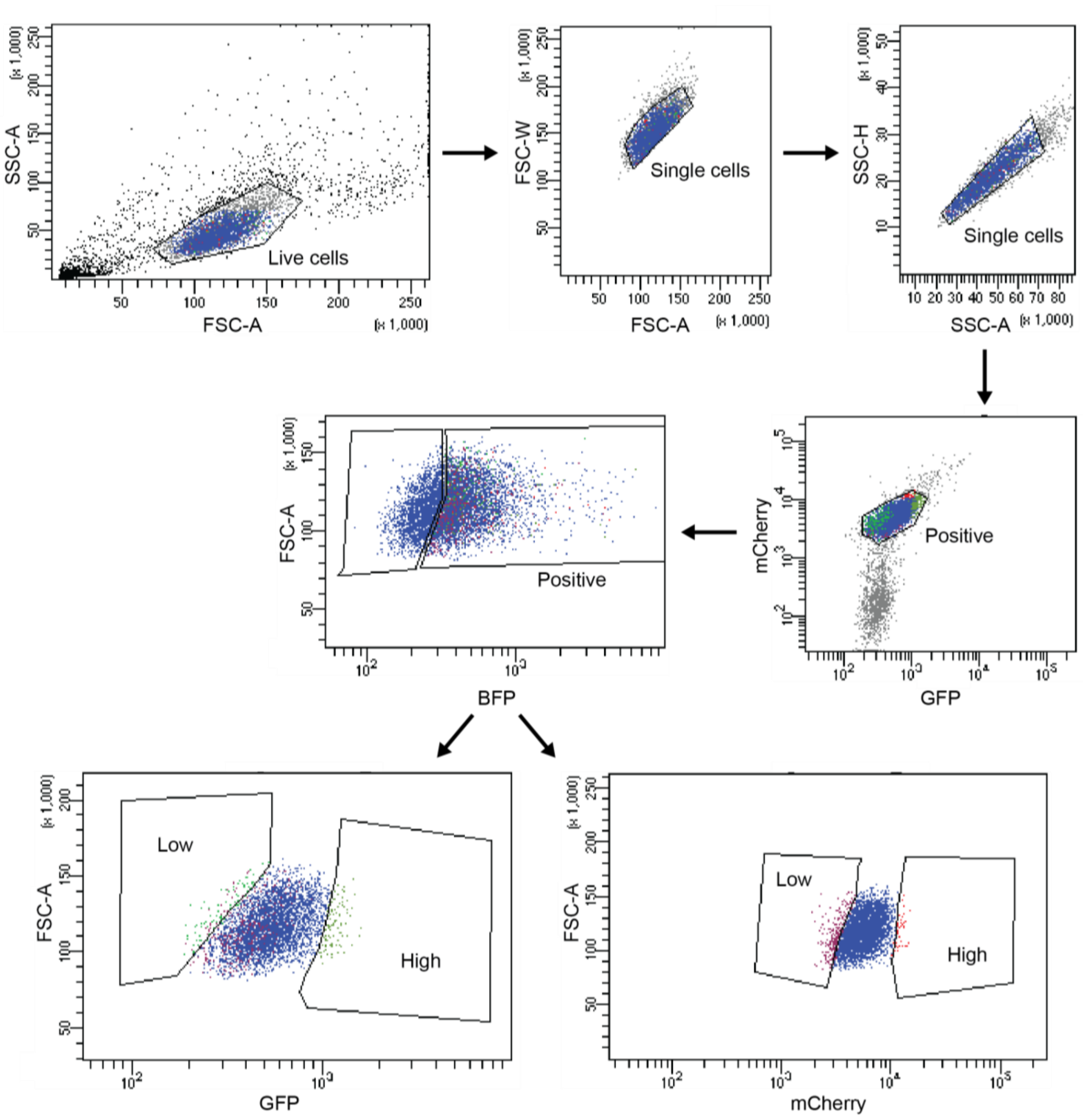

Supplementary information table 1:

Plasmid	Purpose	Reference or source
pLX303_SFFV-MYC-TurboID.TRIM52	Proximity labelling	This study
pLX303_SFFV-MYC-TurboID.EGFP	Proximity labelling	This study
pLX303_SFFV_MYC.mCherry-P2A-OLLAS.EGFP.TRIM52	TRIM52 reporter	This study
pLX303-MYC.mCherry.TRIM52	TRIM52 reporter	This study
pLX303-MYC.mCherry-P2A-3xHA.TRIM52	TRIM52 reporter	This study
pLX303-MYC.mCherry-P2A-3xHA.TRIM52(KtoR)	TRIM52 reporter	This study
pLX303-MYC.mCherry-P2A-3xHA.TRIM52(2KtoR)	TRIM52 reporter	This study
pLX303-MYC.mCherry-P2A-3xHA.TRIM52(5KtoR)	TRIM52 reporter	This study
pLX303-SFFV-TwinStrep.EGFP.TRIM52(R187A)	protein purification	This study
pLX303-SFFV-TwinStrep.EGFP.RING(R187A)	protein purification	This study
pLX303-SFFV-TwinStrep.EGFP.RING(ΔLoop2-R187A)	protein purification	This study
pLX303-SFFV-TwinStrep.EGFP.TRIM52(WT)	protein purification	This study
pLX303-SFFV-TwinStrep.mCherry.TRIM52-hPGK-EBFP	TRIM52 reporter	This study
pLX303-SFFV-TwinStrep.mCherry.TRIM52(ΔLoop2)-hPGK-EBFP	TRIM52 reporter	This study
pLX303-SFFV-TwinStrep.mCherry.RING-hPGK-EBFP	TRIM52 reporter	This study
pLX303-SFFV-TwinStrep.mCherry.RING(ΔLoop2)-hPGK-EBFP	TRIM52 reporter	This study
pLX303-SFFV-TwinStrep.mCherry.RING.Loop2(neut)-hPGK-EBFP	TRIM52 reporter	This study
pLX303-SFFV-TwinStrep.mCherry.RING.Loop2(basic)-hPGK-EBFP	TRIM52 reporter	This study
pLX303-SFFV-TwinStrep.mCherry.Loop2-hPGK-EBFP	TRIM52 reporter	This study
pLX303-SFFV-TwinStrep.mCherry.Loop2(basic)-hPGK-EBFP	TRIM52 reporter	This study
pLX303-SFFV-TwinStrep.mCherry.DAXX-hPGK-EBFP	control	This study
pLX303-SFFV-TwinStrep.mCherry.CY2B-hPGK-EBFP	control	This study
pLX303-SFFV-TwinStrep.mCherry-hPGK-EBFP	control	This study
pcDNA3-His.Ubiquitin	Ubiquitination IP	This study
DualCRISPR-hU6-sgRNA-mU6-sgRNA-EF1as-BFP	Dual sgRNA	de Almeida *et al*.[1]
DualCRISPR-hU6-sgRNA-mU6-sgRNA-EF1as-iRFP	Dual sgRNA	de Almeida M, Hinterndorfer M *et al*.[1]
pLentiCrisprV2-sgRNA-U6-PGK-iRFP670-P2A-Neo	Single sgRNA	This study
PRRL-PBS-U6-sgRNA-EF1as-Thy1-P2A-NeoR	Library sgRNA	Michlits *et al*.[2]

In plasmid names, dashes denote separate elements, points indicate elements fused into a single open reading frame.

Supplementary information table 2:

Gene	Species	Sequence (5' to 3')
ROSA	mouse	AAGATGGGCGGGAGTCTTC
RRM1	human	TACAGTGGGGTCACCACAG
TRIM52_1	human	GAGGGTGTGTGACCCAGCT
TRIM52_2	human	AGCCCTGAAACTCTTCTGTG
UBR4_1	human	GACTGAGGCCACCAGCTG
UBR4_2	human	GGAACCGATTGATATAGCGT
KCMF1_1	human	GGAATGTTTCACCCTGGCCG
KCMF1_2	human	GGGACAAGTAAAAGACTG
BIRC6_1	human	GGATGACTTTGATGGTCCCG
BIRC6_2	human	GCTTGAATTACCCGTTACAG
AAVS1_1	human	CTGTGCCCCGATGCACAC
AAVS1_2	human	GGCGCGTCGCTCGCTCGCTC
HUWE1_1	human	GTAGCCGAGTTAGCAGCG
HUWE1_2	human	GAGATTCCATGATTTCAGAA
TDP2_1	human	AGATTTCCTAGTGATCACTG
TDP2_2	human	GCTCAGAGATGGTTTCAGGT

Supplementary information table 3:

Cell lines and primary cells	Type	Reference or source	Purpose	Media	Supplements
HEK293T	human epithelial	CRL-3216	3xHA-TRIM52/TwinStrep-mCherry-TRIM52 mutants expression	Dulbecco's modified Eagle's medium (DMEM; Sigma-Aldrich, D6429)	10% FBS (Sigma-Aldrich, F7524) and 1% penicillin/streptomycin (Sigma-Aldrich, P4333)
RKO	human colon carcinoma	de Almeida M, Hinterndorfer M et al, 2021	Dox inducible Cas9	RPMI 1640 (Thermo Fisher Scientific, 21875)	10% FBS (Sigma-Aldrich, F7524), L-glutamine (4 mM, Gibco), sodium pyruvate (1 mM, Sigma-Aldrich), and 1% penicillin/streptomycin (Sigma-Aldrich, P4333)

Supplementary information table 4:

Reagent	Abbreviation	Application	Concentration	Manufacturer	Catalogue number
Bafilomycin		Cell culture	400 nM	SantaCruz Biotechnology	sc-201550
Biotin		Cell culture	500 µM	Sigma-Aldrich	B4501-100MG
Capzimin[3]		Cell culture	5 µM	MedChemExpress	HY-110404
Cycloheximide	CHX	Cell culture	200 µg/ml	Sigma-Aldrich	C1988
Doxycycline hyclate	DOX	Cell culture	200-350 ng/ml	Sigma-Aldrich	D9891
Epoxomicin	EPO	Cell culture	10 µM	Gentaur Molecular Products	607-A2606
Etoposide	ETO	Cell culture	5 µM	Sigma-Aldrich	E1383
G418		Cell culture	0.5-1 mg/ml	Sigma-Aldrich	A1720
MG132	MG132	Cell culture	10 µM	Sigma-Aldrich	M7449
MLN-4924	MLN-4924	Cell culture	20 µM	Abcam	ab216470
Puromycin		Cell culture	4 µg/ml	Invivogen	ant-pr-1

Supplementary information table 5:

PCR 1		
Primer name	Direction	Sequence
sgDeepSeq_rev_TGAG	Rv	CTCTTTCCCTACACGACGCTCTTCCGATCTNNNNNNCTCATTCCAGCATAGCTCTTAAAC
sgDeepSeq_rev_TCGA	Rv	CTCTTTCCCTACACGACGCTCTTCCGATCTNNNNNNTCGATTCCAGCATAGCTCTTAAAC
sgDeepSeq_rev_TAGG	Rv	CTCTTTCCCTACACGACGCTCTTCCGATCTNNNNNNCCTATTCCAGCATAGCTCTTAAAC
sgDeepSeq_rev_GTTC	Rv	CTCTTTCCCTACACGACGCTCTTCCGATCTNNNNNNGAACTTCCAGCATAGCTCTTAAAC
sgDeepSeq_rev_GGAT	Rv	CTCTTTCCCTACACGACGCTCTTCCGATCTNNNNNNATCCTTCCAGCATAGCTCTTAAAC
sgDeepSeq_rev_GAGT	Rv	CTCTTTCCCTACACGACGCTCTTCCGATCTNNNNNNACTCTTCCAGCATAGCTCTTAAAC
sgDeepSeq_rev_GAAG	Rv	CTCTTTCCCTACACGACGCTCTTCCGATCTNNNNNNCTTCTTCCAGCATAGCTCTTAAAC
sgDeepSeq_rev_CTTG	Rv	CTCTTTCCCTACACGACGCTCTTCCGATCTNNNNNNCAAGTTCCAGCATAGCTCTTAAAC
sgDeepSeq_rev_CTCA	Rv	CTCTTTCCCTACACGACGCTCTTCCGATCTNNNNNNTGAGTTCCAGCATAGCTCTTAAAC
sgDeepSeq_rev_CGAA	Rv	CTCTTTCCCTACACGACGCTCTTCCGATCTNNNNNNTTCGTTCCAGCATAGCTCTTAAAC
sgDeepSeq_rev_CCTA	Rv	CTCTTTCCCTACACGACGCTCTTCCGATCTNNNNNNTAGGTTCCAGCATAGCTCTTAAAC
sgDeepSeq_rev_CAGA	Rv	CTCTTTCCCTACACGACGCTCTTCCGATCTNNNNNNTCTGTTCCAGCATAGCTCTTAAAC
sgDeepSeq_rev_CAAC	Rv	CTCTTTCCCTACACGACGCTCTTCCGATCTNNNNNNGTTGTTCCAGCATAGCTCTTAAAC
sgDeepSeq_rev_ATCC	Rv	CTCTTTCCCTACACGACGCTCTTCCGATCTNNNNNNGGATTTCCAGCATAGCTCTTAAAC
sgDeepSeq_rev_AGGA	Rv	CTCTTTCCCTACACGACGCTCTTCCGATCTNNNNNNTCCTTTCCAGCATAGCTCTTAAAC
sgDeepSeq_rev_AGCT	Rv	CTCTTTCCCTACACGACGCTCTTCCGATCTNNNNNNAGCTTTCCAGCATAGCTCTTAAAC
sgDeepSeq_rev_AGAC	Rv	CTCTTTCCCTACACGACGCTCTTCCGATCTNNNNNNGTCTTTCCAGCATAGCTCTTAAAC
Fwd1_hybrid_P7_Nras	Fwd	GCATACGAGATAGCTAGCCACC
PCR 2		
Primer name	Direction	Sequence
Rev2_p5_sgDeepSeq	Rv	AATGATACGGCGACCACCGAGATCTACACTCTTTCCCTACACGACGCT
Fwd2_p7_sgDeepSeq	Fwd	CAAGCAGAAGACGGCATACGAGATAGCTAGCCACC

Supplementary information table 6:

Target	Application	Dilution	Conjugate	Manufacturer	Catalogue number	Name	Type
MYC	WB	1:1000		Cell Signaling Technology	5605	c-Myc (D84C12) Rabbit mAb	Primary
Ubiquitin	WB	1:1000		Santa Cruz Biotechnology	sc-8017	Ubiquitin Antibody (P4D1): sc-8017	Primary
Myc Tag	WB	1:5000		Sigma-Aldrich	05-724	Anti-Myc Tag Antibody, clone 4A6	Primary
Ollas	WB	1:50000		Novus	NBP1-06713	OLLAS Epitope Tag Antibody (L2)	Primary
EGFP	WB	1:2000		Abcam	ab6556	Anti-GFP antibody	Primary
ACTIN	WB	1:20000	HRP	Abcam	ab49900	Anti-beta Actin antibody (HRP)	Primary
MYC	FACS	1:100	PE	Cell Signaling Technology	35876	c-Myc/N-Myc (D3N8F) Rabbit mAb (PE Conjugate)	Primary
IgG Isotype Control	IP	1:300		Cell Signaling Technology	2729	Normal Rabbit IgG	Primary
HA	WB	1:1000		Cell Signaling Technology	3724	HA-Tag (C29F4) Rabbit mAb	Primary
HA	WB	1:1000		Cell Signaling Technology	2367	HA-Tag (6E2) Rabbit mAb	Primary
Anti-Rabbit IgG, light chain specific	WB	1:5000	HRP	Jackson ImmunoResearch	211-032-171	Peroxidase IgG Fraction Monoclonal Mouse Anti-Rabbit IgG, light chain specific	Secondary
Vinculin	WB	1:1000		Sigma-Aldrich		V9131	Primary
p-p53, Ser15	WB	1:1000		Cell Signaling Technology	9284		Primary
p-p53	WB	1:1000		Cell Signaling Technology	2524	1C12	Primary
HRP-β-actin	WB	1:20000	HRP	Abcam	ab49900	AC-15	Primary
HRP anti-rabbit IgG	WB	1:3500	HRP	Cell Signaling Technology	7074		Secondary
HRP anti-mouse IgG	WB	1:3500	HRP	Cell Signaling Technology	7076		Secondary
TRIM52	WB	1:500		Santa Cruz Biotechnology	Sc-398954	TRIM52 (A-4): sc-398954	Primary
LC3B	WB	1:1000		Cell Signaling Technology	3868	LC3B (D11) XP® Rabbit mAb	Primary
HUWE1	WB	1:1000		Bethyl Laboratories	A300-486A	Rabbit anti-Lasu1/Ureb1 Antibody (HUWE1)	Primary
BIRC6	WB	1:1000		Cell Signaling Technology	8756	BIRC6 (D8B5) Rabbit mAb	Primary
UBR4	WB	1:1000		Abcam	ab86738	Anti-UBR4/p600 antibody (ab86738)	Primary
KCMF1	WB	1:500		Sigma-Aldrich	HPA030383	Anti-KCMF1 antibody	Primary
GFP	WB	1:1000		Abcam	ab6556	Anti-GFP antibody	Primary
Biotin	WB	1:1000	HRP	Invitrogen	434323	Streptavidin-HRP	Primary
FLAG	WB	1:1000		Sigma-Aldrich	F1804	Monoclonal ANTI-FLAG® M2 antibody	Primary

Supplementary Methods

Western blot analysis

Cells were lysed in 1x disruption buffer or RIPA lysis buffer supplemented with 1% SDS and 25 U/ml benzonase. Lysates were rotated for 30 min. at 4 °C and then centrifuged at 18,500 x g for 10 min. at 4 °C. Supernatants were transferred to new tubes and protein concentrations were determined by BCA Protein Assay Kit (Thermo Fisher Scientific, 23225). Between 20-50 µg of protein per sample was mixed with Laemmli sample buffer (62.5 mM Tris-HCl (pH 6.8), 5.8% glycerol, 2% SDS and 1.7% β-mercaptoethanol), and boiled for 10 min. Proteins were loaded on 10% SDS polyacrylamide gels, or alternatively 4–20% Mini-PROTEAN TGX Stain-Free (BioRad, 4568094) or 3-8% NuPage gels (Invitrogen, EA03785BOX) to probe for high molecular weight proteins. Proteins were separated by SDS-PAGE using Tris-Glycine (25 mM Tris, 192 mM glycine, 0.1% SDS) or Tris-Acetate (2.5 mM Tricine, 2.5 mM Tris, 0.05% SDS) SDS running buffer, respectively. Proteins were blotted on PVDF membranes at 4 °C for 1 h and 15 min. at 300 mA in Towbin buffer (25 mM Tris pH 8.3, 192 mM glycine and 20% ethanol). Membranes were blocked in 5% BSA in PBS-T for 1 h. at RT, and subsequently incubated with primary antibodies diluted in 5% BSA overnight at 4 °C. The next day, membranes were washed three times for 5 min. each with PBS-0.05%-Tween20 and incubated with HRP-coupled secondary antibodies in 5% skimmed milk for 1 h. at RT and imaged with the ChemiDoc Imaging System (Bio-Rad). Relative protein levels were quantified using Image Lab software (Bio-Rad). The antibodies used in this study are listed in Table S6.

Sample preparation for nLC-MS/MS analysis

Beads were resuspended in 96 µl 50 mM ammonium bicarbonate and reduced with 2 µl of 50mM TCEP for 30 min. at RT before adding 2 µl of 200 mM MMTS and incubating for 30 min. at RT in the dark. Afterwards, samples were digested with 300 ng trypsin (Trypsin Gold, Promega; V5280) at 37 °C overnight. Supernatants were transferred to new tubes and digests were stopped by the addition of trifluoroacetic acid (TFA) to a final concentration of 0.5 %, and peptides were desalted using C18 Stagetips[4]. Half of the trypsin digests were dried in a SpeedVac and resuspended in 100 mM Tris-HCl (pH 8.5). Samples were further digested with 50 ng chymotrypsin at 25°C for 5 h. The digests were stopped by the addition of trifluoroacetic acid (TFA) to a final concentration of 0.5 %, and the peptides were desalted using C18 Stagetips[4].

Liquid chromatography-mass spectrometry data acquisition and analysis

For the identification of ubiquitinated residues, peptides were separated on an Ultimate 3000 RSLC nano-flow chromatography system (Thermo Fisher Scientific), using a pre-column for sample loading (Acclaim PepMap C18, 2 cm × 0.1 mm, 5 μm, Thermo Fisher Scientific), and a C18 analytical column (Acclaim PepMap C18, 50 cm × 0.75 mm, 2 μm, Thermo Fisher Scientific), applying a segmented linear gradient from 2% to 35% and finally 80% solvent B (80 % acetonitrile, 0.1 % formic acid; solvent A 0.1 % formic acid) at a flow rate of 230 nl/min over 60 min. Eluting peptides were analyzed on an Exploris 480 Orbitrap mass spectrometer (Thermo Fisher Scientific) coupled to the column with a FAIMS pro ion-source (Thermo Fisher Scientific) using coated emitter tips (PepSep, MSWil) with the following settings: the mass spectrometer was operated in DDA mode with two FAIMS compensation voltages (CV) set to -35, -45, -60 or -75 and 0.8 s cycle time per CV. The survey scans were obtained in a mass range of 350-1500 m/z, at a resolution of 60k at 200 m/z, and a normalized AGC target at 100%. The most intense ions were selected with an isolation width of 1.2 m/z, fragmented in the HCD cell at 28% collision energy, and the spectra recorded for max. 100 ms at a normalized AGC target of 200% and a resolution of 30k. Peptides with a charge of +2 to +6 were included for fragmentation, the peptide match feature was set to preferred, the exclude isotope feature was enabled, and selected precursors were dynamically excluded from repeated sampling for 20 seconds. MS raw data split for each CV using FreeStyle 1.7 (Thermo Fisher Scientific), were analyzed using the MaxQuant software package (version 2.1.0.0)[5] with the Uniprot human reference proteome (version 2022_01, www.uniprot.org), target protein sequences, as well as a database of most common contaminants. The search was performed with trypsin/chymotrypsin specificity and a maximum of two or four missed cleavages at a protein and peptide spectrum match false discovery rate of 1%. MMTS of cysteine residues was set as fixed, GlyGly(K), oxidation of methionine, and N-terminal acetylation as variable modifications - all other parameters were left at default. The mass spectrometry proteomics data have been deposited to the ProteomeXchange Consortium via the PRIDE partner repository[6] with the dataset identifier PXD051295 .

For TurboID proximity labelling analysis, peptides were separated on an Ultimate 3000 RSLC Nano-flow chromatography system (Thermo Fisher Scientific), using a pre-column for sample loading (Acclaim PepMap C18, 2 cm × 0.1 mm, 5 μm, Thermo Fisher Scientific), and a C18 analytical column (Acclaim PepMap C18, 50 cm × 0.75 mm, 2 μm, Thermo Fisher Scientific), applying a segmented linear gradient from 2% to 35% and finally 80% solvent B (80 % acetonitrile, 0.1 % formic acid; solvent A 0.1 % formic acid) at a flow rate of 230 nl/min over 120 min. Eluting peptides were analyzed on an Exploris 480 Orbitrap mass spectrometer (Thermo Fisher Scientific) coupled to the column with a FAIMS pro ion-source (Thermo Fisher Scientific) using coated emitter tips (PepSep, MSWil) with the following settings: the mass spectrometer was operated in DDA mode with two FAIMS compensation voltages (CV) set to

-45 or -60 and 1.5 s cycle time per CV. The survey scans were obtained in a mass range of 350-1500 m/z, at a resolution of 60k at 200 m/z, and a normalized AGC target at 100%. The most intense ions were selected with an isolation width of 1 m/z, fragmented in the HCD cell at 28% collision energy, and the spectra recorded for max. 50 ms at a normalized AGC target of 100% and a resolution of 15k. Peptides with a charge of +2 to +6 were included for fragmentation, the peptide match feature was set to preferred, the exclude isotope feature was enabled, and selected precursors were dynamically excluded from repeated sampling for 45 seconds. MS raw data split for each CV using FreeStyle 1.7 (Thermo Fisher Scientific), were analyzed using the MaxQuant software package (version 1.6.17.0)[5] with the Uniprot human reference proteome (version 2021_03, www.uniprot.org), as well as a database of most common contaminants. The search was performed with full trypsin specificity and a maximum of two missed cleavages at a protein and peptide spectrum match false discovery rate of 1%. Carbamidomethylation of cysteine residues was set as fixed, oxidation of methionine, phosphorylation (STY) and N-terminal acetylation as variable modifications. For label-free quantification the "match between runs" only within the sample batch and the LFQ function were activated - all other parameters were left at default. MaxQuant output tables were further processed in R 4.2.1 (https://www.R-project.org) using Cassiopeia_LFQ (https://github.com/moritzmadern/Cassiopeia_LFQ). Reverse database identifications, contaminant proteins, protein groups identified only by a modified peptide, protein groups with less than two quantitative values in one experimental group, and protein groups with less than 2 razor peptides were removed for further analysis. Missing values were replaced by randomly drawing data points from a normal distribution model on the whole dataset (data mean shifted by -1.8 standard deviations, a width of the distribution of 0.3 standard deviations). The mass spectrometry proteomics data have been deposited to the ProteomeXchange Consortium via the PRIDE partner repository with the dataset identifier PXD051272.

Protein purification

Baculovirus encoding the respective TwinStrep tagged EGFP-TRIM52 constructs (TRIM52-WT, TRIM52-R187A, RING-R187A, RING ΔLoop2) were added to 500 mL of $1x10^6$ mL^{-1} Trichoplusia ni High Five (Accession: CVCL_C190) insect cells at a ratio of 1:100, grown in ESF921 serum-free media. Cell growth continued at 27 °C at 100 rpm for three days, after which cells were pelleted at 500 x g for 10 min. and resuspended in 25 mM HEPES pH 7.5, 300 mM KCl before flash freezing. For purification, cells were thawed and mixed with two mini c0mplete EDTA free protease inhibitor tablets (Roche) and bezonase (Molecular Biology Services, IMP). Cells were dounced 10 times with a glass douncer leading to mechanical lysis, and supernatant was collected after a 45 min, centrifugation step at 19000 rpm. The sample

was applied to a 1 mL StrepTrap column (Cytiva), washed, and eluted in the same buffer with 3 mM d-desthiobiotin. Peak fractions were pooled, concentrated, and loaded onto a Superdex 200 10/300 (Cytiva) column in 25 mM HEPES pH 7.5, 300 mM KCl via a 1 mL loop. Fractions and purity were chosen and assessed by SDS-PAGE and the resulting protein was concentrated and frozen.

Recombinant human BIRC6, UBCH5B, UBA1, UBA6, Ubiquitin, DyLight800-labeled Ubiquitin were purified as previously described[7,8]. Human HUWE1 was codon-optimized, synthesized in fragments and assembled into a GoldenBac pGBdest vector via a BsaI-GoldenGate reaction cloning[9]. The construct contains a His8-tag, a rigid enhancer linker (AEAAAKEAAAKEAAAKEAAAKALEAEAAAKEAAAKEAAAKEAAAKA) followed by a TEV cleavage site. Human UBR4 was cloned by the same approach, but instead with a C-terminal Strep tag which is separated from the protein via a 3C cleavage site. Expression plasmids were transformed into the DH10 MultiBac cells for bacmid generation. *Spodoptera frugiperda* (Sf9) cells were cultured in ESF921 serum-free growth medium (Expression Systems) and transfected with the bacmids for virus production, which was quantified using yellow fluorescent protein signal. HUWE1 and UBR4 were then expressed in *Trichoplusia ni* High-Five insect cells (Thermo Fisher Scientific) at a density of 1.5 x10^6. The cells were inoculated with a 1:70 dilution from the V1 stock for 92 h. at 21°C in Insect Xpress Protein-free Insect Cell Medium (Lonza) supplemented with GlutaMAX (GIBCO) and Pen/Strep amphotericin B (Lonza). Cells were harvested by centrifugation at 700 x g, washed in PBS, flash-frozen and stored at -70°C.

For HUWE1 purification, the cell pellet was thawed and resuspended in 50 mM HEPES pH 7.5, 300 mM NaCl, 0.5 mM TCEP, 20 mM imidazole, supplemented with Complete EDTA-free Protease inhibitor (Roche) and 20 µl benzonase (IMP Molecular Biology Service) and lysed using a douncer. The supernatant was separated by centrifugation at 40,000 x g. and the soluble fraction loaded on a 5 ml HisTrap HP column (Cytiva). The column was pre-equilibrated in the lysis buffer using an Äkta Pure 25 system (Cytiva). The column was washed with 10 CVs of the same buffer, followed by 7 CVs of buffer supplemented with 35 mM imidazole, and then eluted with 300 mM imidazole. The protein was cleaved with TEV protease and simultaneously dialyzed overnight to remove the imidazole. The cleaved protein was then reapplied to HisTrap HP column in 20 mM imidazole and flow through collected and concentrated to 1.5 ml. Following that, the protein was applied to a Superose 6 16/60 column (Cytiva) equilibrated in 50 mM HEPES pH 7.5, 150 mM NaCl, 0.5 mM TCEP and HUWE1-containing fractions were pooled and concentrated. The protein purity was assessed by SDS-PAGE and the concentration was estimated by absorption at A280 nm using an extinction coefficient of 251,770 M^{-1} cm^{-1}.

For UBR4 purification, the same lysis procedure was used except with phosphate buffered saline (PBS), and 0.5 mM TCEP at pH 7.4 as the buffer. The soluble lysate was loaded on a 5 ml StrepTrap HP column (Cytiva) equilibrated in PBS, washed with the same buffer and eluted with 2.5 mM desthiobiotin. Eluted UBR4 was then applied to a Resource Q column (Cytiva) equilibrated in PBS for additional purification via anion exchange chromatography using a 250 to 500 mM NaCl gradient. UBR4-containing fractions were pooled and concentrated. The concentration was measured by absorption at 280 nm using a calculated extinction coefficient of 472,140 $M^{-1}cm^{-1}$.

The codon-optimized UBE2A (RAD6) gene fragment was synthesized by Twist Bioscience. The NEB HiFi assembly kit (New England Biolabs) was used to clone the gene fragment into a pET29b expression vector, which also contained an N-terminal His6-tag and TEV cleavage site. The protein was expressed from *E. coli* BL21 (DE3) cells using IPTG induction at 20 °C overnight. Cells were resuspended in buffer containing 50 mM Tris (pH 7.5), 150 mM NaCl, 10 mM imidazole, supplemented with complete EDTA-free protease inhibitor cocktail. Cells were lysed by sonication and the supernatant was separated by centrifugation at 18,500x g for 20 min at 4 °C. The soluble fraction was incubated with Ni-NTA resin (Qiagen) for 1 h at 4 °C with mild agitation. Ni-NTA resin was washed before elution with 150 mM imidazole. UBE2A was additionally purified by SEC using a Superdex 75 16/600 column (Cytiva) equilibrated in 50 mM Tris, 150 mM NaCl and 0.5 mM TCEP, pH 7.5.

Ontology analysis

Differentially enriched GO terms were obtained using online Enrichr ontology analysis tool[10–12] with GO Biological processes 2023 library or Reactome 2022 library [13,14] as a reference. The enriched terms are plotted based on their adjusted p-value and the number of genes within each GO Biological Processes 2023 or Reactome 2022 term. The adjusted p-value is calculated using the Benjamini Hoochberg method for multiple hypotheses testing correction.

Supplementary References

1. de Almeida, M. *et al.* AKIRIN2 controls the nuclear import of proteasomes in vertebrates. *Nature* **599**, 491–496 (2021).
2. Michlits, G. *et al.* Multilayered VBC score predicts sgRNAs that efficiently generate loss-of-function alleles. *Nat. Methods* **17**, 708–716 (2020).
3. Li, J. *et al.* Capzimin is a potent and specific inhibitor of proteasome isopeptidase Rpn11. *Nat. Chem. Biol.* **13**, 486–493 (2017).
4. Rappsilber, J., Mann, M. & Ishihama, Y. Protocol for micro-purification, enrichment, pre-fractionation and storage of peptides for proteomics using StageTips. *Nat. Protoc.* **2**, 1896–1906 (2007).
5. Tyanova, S., Temu, T. & Cox, J. The MaxQuant computational platform for mass spectrometry-based shotgun proteomics. *Nat. Protoc.* **11**, 2301–2319 (2016).
6. Perez-Riverol, Y. *et al.* The PRIDE database and related tools and resources in 2019: improving support for quantification data. *Nucleic Acids Res.* **47**, D442–D450 (2019).
7. Ehrmann, J. F. *et al.* Structural basis for regulation of apoptosis and autophagy by the BIRC6/SMAC complex. *Science* **379**, 1117–1123 (2023).
8. Hunkeler, M., Jin, C. Y. & Fischer, E. S. Structures of BIRC6-client complexes provide a mechanism of SMAC-mediated release of caspases. *Science* **379**, 1105–1111 (2023).
9. Neuhold, J. *et al.* GoldenBac: a simple, highly efficient, and widely applicable system for construction of multi-gene expression vectors for use with the baculovirus expression vector system. *BMC Biotechnol.* **20**, 26 (2020).
10. Chen, E. Y. *et al.* Enrichr: interactive and collaborative HTML5 gene list enrichment analysis tool. *BMC Bioinformatics* **14**, 128 (2013).
11. Kuleshov, M. V. *et al.* Enrichr: a comprehensive gene set enrichment analysis web server 2016 update. *Nucleic Acids Res.* **44**, W90–W97 (2016).
12. Xie, Z. *et al.* Gene Set Knowledge Discovery with Enrichr. *Curr. Protoc.* **1**, e90 (2021).
13. The Gene Ontology Consortium *et al.* The Gene Ontology knowledgebase in 2023. *Genetics* **224**, iyad031 (2023).
14. Ashburner, M. *et al.* Gene Ontology: tool for the unification of biology. *Nat. Genet.* **25**, 25–29 (2000).
