# Supplementary material for: TRIM52 maintains cellular fitness and is under tight proteolytic control by multiple giant E3 ligases
Source: Nat Commun. 2025 Apr 24;16:3894. doi: 10.1038/s41467-025-59129-y (PMC12022042; doi:10.1038/s41467-025-59129-y)
Supplement: Supplementary file 2 — Description of Additional Supplementary Files [file 41467_2025_59129_MOESM2_ESM.pdf]

Description of Additional Supplementary Files

File Name: Supplementary Data 1
Description: MaGeCK data for genetic modifier screen presented in Figures 1e-f.

File Name: Supplementary Data 2
Description: TurboID nLC-MS/MS data presented in Figures 2a-b and 5d-e.

File Name: Supplementary Data 3
Description: Gene Otology analysis data presented in Supplementary Figures 2h-j and Supplementary Figure 6h.

File Name: Supplementary Data 4
Description: nLC-MS/MS identification of ubiquitination sites data presented in Figure 3h.

File Name: Supplementary Data 5
Description: MaGeCK data for genetic TRIM52 degrader screen presented in Figures 4c-d and Supplementary Figure 5b.

File Name: Supplementary Data 6
Description: Combinations of recombinant proteins used in in vitro ubiquitination experiments presented in Figures 6a-f and Supplementary Figures 7a-f.
