# Supplementary material for: TRIM52 maintains cellular fitness and is under tight proteolytic control by multiple giant E3 ligases
Source: Nat Commun. 2025 Apr 24;16:3894. doi: 10.1038/s41467-025-59129-y (PMC12022042; doi:10.1038/s41467-025-59129-y)
Supplement: Supplementary file 9 — Reporting Summary [file 41467_2025_59129_MOESM9_ESM.pdf]

nature portfolio

Corresponding author(s): Gijs A. Versteeg

Last updated by author(s): Dec 17, 2024

Reporting Summary

Nature Portfolio wishes to improve the reproducibility of the work that we publish. This form provides structure for consistency and transparency in reporting. For further information on Nature Portfolio policies, see our Editorial Policies and the Editorial Policy Checklist.

Statistics

For all statistical analyses, confirm that the following items are present in the figure legend, table legend, main text, or Methods section.

n/a	Confirmed	
☐	☒	The exact sample size (*n*) for each experimental group/condition, given as a discrete number and unit of measurement
☐	☒	A statement on whether measurements were taken from distinct samples or whether the same sample was measured repeatedly
☐	☒	The statistical test(s) used AND whether they are one- or two-sided<br>*Only common tests should be described solely by name; describe more complex techniques in the Methods section.*
☒	☐	A description of all covariates tested
☒	☐	A description of any assumptions or corrections, such as tests of normality and adjustment for multiple comparisons
☐	☒	A full description of the statistical parameters including central tendency (e.g. means) or other basic estimates (e.g. regression coefficient) AND variation (e.g. standard deviation) or associated estimates of uncertainty (e.g. confidence intervals)
☐	☒	For null hypothesis testing, the test statistic (e.g. *F*, *t*, *r*) with confidence intervals, effect sizes, degrees of freedom and *P* value noted<br>*Give P values as exact values whenever suitable.*
☒	☐	For Bayesian analysis, information on the choice of priors and Markov chain Monte Carlo settings
☒	☐	For hierarchical and complex designs, identification of the appropriate level for tests and full reporting of outcomes
☒	☐	Estimates of effect sizes (e.g. Cohen's *d*, Pearson's *r*), indicating how they were calculated

Our web collection on statistics for biologists contains articles on many of the points above.

Software and code

Policy information about availability of computer code

Data collection	No newly developed software or code was used
Data analysis	No newly developed software or code was used. Statistical analysis on TurboID data was conducted with the limma v.3.54.2 package in R.

For manuscripts utilizing custom algorithms or software that are central to the research but not yet described in published literature, software must be made available to editors and reviewers. We strongly encourage code deposition in a community repository (e.g. GitHub). See the Nature Portfolio guidelines for submitting code & software for further information.

Data

Policy information about availability of data

All manuscripts must include a data availability statement. This statement should provide the following information, where applicable:

- Accession codes, unique identifiers, or web links for publicly available datasets
- A description of any restrictions on data availability
- For clinical datasets or third party data, please ensure that the statement adheres to our policy

All data generated or analysed during this study are included in the manuscript and supporting files. Source data are provided with this paper. The genetic screen data generated in this study are provided in the Source Data file. The mass-spectrometry data generated in this study have been deposited in the PRIDE database under accession codes PXD051295 (https://www.ebi.ac.uk/pride/archive/projects/PXD051295) and PXD051272 (https://www.ebi.ac.uk/pride/archive/projects/PXD051272).

Research involving human participants, their data, or biological material

Policy information about studies with human participants or human data. See also policy information about sex, gender (identity/presentation), and sexual orientation and race, ethnicity and racism.

Reporting on sex and gender	does not apply
Reporting on race, ethnicity, or other socially relevant groupings	does not apply
Population characteristics	does not apply
Recruitment	does not apply
Ethics oversight	does not apply

Note that full information on the approval of the study protocol must also be provided in the manuscript.

Field-specific reporting

Please select the one below that is the best fit for your research. If you are not sure, read the appropriate sections before making your selection.

☒ Life sciences ☐ Behavioural & social sciences ☐ Ecological, evolutionary & environmental sciences

For a reference copy of the document with all sections, see nature.com/documents/nr-reporting-summary-flat.pdf

Life sciences study design

All studies must disclose on these points even when the disclosure is negative.

Sample size	Experiments were performed in biological triplicates, unless indicated otherwise in the figure legends.
Data exclusions	No data were excluded from analysis
Replication	Experiments were reproduced at least two times independently with the exceptions: of FACS-based CRIPIR-iCas9 genetic screens and Turbo-ID proximity labeling as these results were independently validated using additional experiments; in-vitro ubiquitination assay with 3xRA BIRC6 mutant was also not reproduced due to the low availability of the purified protein; lc-MS/MS identification of ubiquitinated residues on TRIM52 purified from cell lysate.
Randomization	Does not apply;
Blinding	does not apply;

Reporting for specific materials, systems and methods

We require information from authors about some types of materials, experimental systems and methods used in many studies. Here, indicate whether each material, system or method listed is relevant to your study. If you are not sure if a list item applies to your research, read the appropriate section before selecting a response.

Materials & experimental systems

n/a	Involved in the study	
☐	☒	Antibodies
☐	☒	Eukaryotic cell lines
☒	☐	Palaeontology and archaeology
☒	☐	Animals and other organisms
☒	☐	Clinical data
☒	☐	Dual use research of concern
☒	☐	Plants

Methods

n/a	Involved in the study	
☒	☐	ChIP-seq
☐	☒	Flow cytometry
☒	☐	MRI-based neuroimaging

Antibodies

Antibodies used	All antibodies were purchased commercially, and are all described in manuscript and associated files.

Antibodies used

Target Application Dilution Manufacturer Catalogue number
MYC WB 1:1000 Cell Signaling Technology 5605
Ubiquitin WB 1:1000 Santa Cruz Biotechnology sc-8017
Myc Tag WB 1:5000 Sigma-Aldrich 05-724
Ollas WB 1:50000 Novus NBP1-06713
EGFP WB 1:2000 Abcam ab6556
ACTIN WB 1:20000 Abcam ab49900
MYC FACS 1:100 Cell Signaling Technology 35876
IgG Isotype Control IP 1:300 Cell Signaling Technology 2729
HA WB 1:1000 Cell Signaling Technology 3724
HA WB 1:1000 Cell Signaling Technology 2367
Anti-Rabbit IgG, light chain specific WB 1:5000 Jackson ImmunoResearch 211-032-171
Vinculin WB 1:1000 Sigma-Aldrich V9131
p-p53, Ser15 WB 1:1000 Cell Signaling Technology 9284
p-p53 WB 1:1000 Cell Signaling Technology 2524
HRP-β-actin WB 1:20000 Abcam ab49900
HRP anti-rabbit IgG WB 1:3500 Cell Signaling Technology 7074
HRP anti-mouse IgG WB 1:3500 Cell Signaling Technology 7076
TRIM52 WB 1:500 Santa Cruz Biotechnology Sc-398954
LC3B WB 1:1000 Cell Signaling Technology 3868
HUWE1 WB 1:1000 Bethyl Laboratories A300-486A
BIRC6 WB 1:1000 Cell Signaling Technology 8756
UBR4 WB 1:1000 Abcam ab86738
KCMF1 WB 1:500 Sigma-Aldrich HPA030383
GFP WB 1:1000 Abcam ab6556
Biotin WB 1:1000 Invitrogen 434323
FLAG WB 1:1000 Sigma-Aldrich F1804

Validation

Antibodies essential for the study (such as detecting TRIM52) were validated in cell lines in which the gene expressing the targeted protein was knocked out (Figure 1b).

Eukaryotic cell lines

Policy information about cell lines and Sex and Gender in Research

Cell line source(s)

All cell lines are described in the manuscript and associated files.

Cell lines and primary cells Type Reference or source
HEK293T human epithelial CRL-3216
RKO human colon carcinoma de Almeida M, Hinterndorfer M et al, 2021

Authentication

Cell lines used in this study were authenticated by STR

Mycoplasma contamination

Cell lines were routinely tested for mycoplasma

Commonly misidentified lines
(See ICLAC register)

does not apply

Plants

Seed stocks

does not apply

Novel plant genotypes

does not apply

Authentication

does not apply

Flow Cytometry

Plots

Confirm that:

☒ The axis labels state the marker and fluorochrome used (e.g. CD4-FITC).

☒ The axis scales are clearly visible. Include numbers along axes only for bottom left plot of group (a 'group' is an analysis of identical markers).

☐ All plots are contour plots with outliers or pseudocolor plots.

☐ A numerical value for number of cells or percentage (with statistics) is provided.

Methodology

Sample preparation	Sample preparation is described in the manuscript and its associated files.
Instrument	Samples were collected using FACS Aria III cell sorter operated by BD FACSDiva software (v8.0), Bio-Rad ZE5 Cell Analyzer operated by Everest software, BD LSRFortessa Cell Analyzer operated by FACSDiva software.
Software	Samples were analyzed and plotted using FlowJo v10.8.1 software.
Cell population abundance	Live-sorted samples were routinely checked for purity using negative as well as positive controls, and only analyzed when the appropriate cell population was present.
Gating strategy	Gating strategy for the genetic screen is described in the materials and methods section. Cells were gated for live, single as well as relevant fluorophore-positive populations using negative as well as positive control samples. In its current form, the manuscript does not have detailed plots that report the gating strategies, however this can be provided if needed

☒ Tick this box to confirm that a figure exemplifying the gating strategy is provided in the Supplementary Information.
